# Supplementary material for: MetaRibo-Seq measures translation in microbiomes
Source: Nat Commun. 2020 Jun 29;11:3268. doi: 10.1038/s41467-020-17081-z (PMC7324362; doi:10.1038/s41467-020-17081-z)
Supplement: Supplementary file 10 — Supplementary Data 7 [file 41467_2020_17081_MOESM10_ESM.zip › File2/Confidence_High_Taxonomy/421222_out.krona.html]

Javascript must be enabled to view this page.

members
magnitude
magnitudeUnassigned
count
unassigned
taxon
rank

421222\_out

48

48
2
superkingdom

976
phylum
48

class
200643
48

48
order
171549

815
family
48

genus
816
48

2

SRS048870\_contig\_number\_4254SRS078419\_contig\_number\_11366
671267
species

2

SRS045713\_contig\_number\_12919SRS077127\_contig\_number\_4206
1263042
species


SRS016018\_contig\_number\_4057SRS016381\_contig\_number\_2545SRS018049\_contig\_number\_472SRS018541\_contig\_number\_5108SRS018984\_contig\_number\_5458SRS019286\_contig\_number\_5301SRS042628\_contig\_number\_34867SRS043001\_contig\_number\_1900SRS046717\_contig\_number\_3696SRS048981\_contig\_number\_1868SRS058070\_contig\_number\_14258SRS058770\_contig\_number\_26891SRS075021\_contig\_number\_342SRS077024\_contig\_number\_10550SRS098717\_contig\_number\_12872SRS101268\_contig\_number\_127SRS1041038\_contig\_number\_12933SRS1041112\_contig\_number\_2037SRS104636\_contig\_number\_15401SRS1055034\_contig\_number\_42
species
357276
20


SRS023583\_contig\_number\_4741
1263052
species
1

2
species
818

SRS018133\_contig\_number\_2133SRS140645\_contig\_number\_655

1

SRS149879\_contig\_number\_18469
species
371601


SRS149784\_contig\_number\_24471
2292943
species
1

821
species

SRS023715\_contig\_number\_25457SRS045195\_contig\_number\_7147SRS053649\_contig\_number\_20786SRS148874\_contig\_number\_13812SRS893300\_contig\_number\_10331
5


SRS013940\_contig\_number\_5988
species
329854
1

28116
species

SRS015264\_contig\_number\_13473SRS054590\_contig\_number\_232SRS142712\_contig\_number\_4855
3


SRS014613\_contig\_number\_20125
162156
species
1

6

SRS048060\_contig\_number\_1022SRS064232\_contig\_number\_1622SRS064276\_contig\_number\_17832SRS1041145\_contig\_number\_24370SRS1054691\_contig\_number\_contig-100\_155.111284SRS1055067\_contig\_number\_10049
2292935
species


SRS019161\_contig\_number\_9093
2292944
species
1


SRS024663\_contig\_number\_4079
species
820
1

1

SRS012849\_contig\_number\_33591
species
310297
